# S1-2 Findings from the children and adolescent working group project on whole systems approaches to whole school physical activity promotion across Europe

**DOI:** 10.1093/eurpub/ckad133.005

**Published:** 2023-09-11

**Authors:** Jade Morris

**Affiliations:** Bradford University

## Abstract

This talk will focus on a working group project investigating the research, policy and practice needs related to co-developing a whole-school system approach to physical activity (PA).

The World Health Organization (2018) calls for all countries to implement a whole-system approach and positions school as an opportune site for increasing PA opportunities for children. Yet, current research reveals that most whole-school approaches fail to create impactful and sustainable changes. Consistent limitations include a focus on short-term initiatives that fail to address the many ingredients required to promote systems change through addressing organisational structure and culture within and beyond school settings. While contemporary guidance provides an in-depth understanding of “what” factors to address within the school, the system still lacks an understanding of “how” to address each factor required to create impactful and sustainable change. It is essential that the implementation of the different ingredients that form the school system are co-developed with multi-stakeholders from within and beyond the school environment.

**Project description:**

To establish the research, policy and practice needs related to co-developing a whole-school systems approach to PA, this project includes three stages.

First, we drew on an extensive European network to identify and categorise stakeholders involved in influencing and/or operationalising whole-school PA across the region.

Second, we will conduct a needs analysis to systematically identify training needs. We will conduct six focus groups across Europe to gain diverse stakeholders perspectives on the required training needs aligned to capabilities, opportunities, and motivation (COM-B). Finally, we will be using the aforementioned insights to create a policy briefing on whole-school systems-based PA training needs. We will conduct two virtual stakeholder events to consult on the findings and gather further insights, offering contextual different within and between countries.

**Conclusion:**

The policy briefing will provide recommendations for working across national contexts relating to school-based PA, providing guidance for policymakers and practitioners. We anticipate these insights will promote the development of bottom-up and top-down approaches to create ‘systems’ change within the school context.

**Funding:**

This project is supported by the WHO European Office for Prevention and Control of Noncommunicable Diseases.

